# Feasibility of a novel slim gastroscope for endoscopic submucosal dissection: A case series (with video)

**DOI:** 10.1002/deo2.70139

**Published:** 2025-05-06

**Authors:** Marina Kim, Yohei Minato, Darshan Parekh, Hideyuki Chiba, Ryoju Negishi, Shunya Takayanagi, Kohei Ono, Yuki Kano, Yoshiaki Kimoto, Nao Takeuchi, Shinya Nagae, Ken Ohata

**Affiliations:** ^1^ Gastroenterology, NTT Medical Center Tokyo Tokyo Japan; ^2^ Gastroenterology and Hepatology, Saint Louis University Saint Louis USA; ^3^ Advanced Endoscopy Mumbai Institute of Gastroenterology Mumbai India; ^4^ Gastroenterology, Omori Red Cross Hospital Tokyo Japan

**Keywords:** endoscopes, endoscopic submucosal dissection, feasibility study, gastrointestinal, gastrointestinal neoplasm, pharynx

## Abstract

Endoscopic submucosal dissection has transformed early‐stage gastrointestinal tumor treatment. This series showcases a novel slim gastroscope's efficacy in tackling challenging lesions.

Retrospective analysis of 17 patients undergoing endoscopic submucosal dissection using the novel slim gastroscope for pharyngeal, esophageal, gastric, duodenal, and rectal lesions at our tertiary care center between November 2022 and July 2023. The slim scope has an outer diameter of 7.9 mm, an accessory channel diameter of 3.2 mm, and a downward bending angle of 160 degrees. Primary outcomes were en‐bloc and R0 resection rate and secondary outcomes were procedure time, adverse events, and specimen/defect size.

100% successful en bloc and R0 resections were achieved with no significant adverse events. The median lesion size was 15 mm (2–40 mm), and the median procedure time was 30 min (5–105 min). Various strategies, including multiple tunnels, pocket creation,​ and endoscopic intermuscular dissection, were employed.

The novel slim gastroscope is feasible for endoscopic submucosal dissection in many locations including the pharynx and duodenum and in certain complex lesions (large gastric lesions, rectal lesions with deep submucosal invasion, and circumferential esophageal lesions). This warrants further investigation through larger comparative studies to validate its efficacy and safety in a broader patient population.

## INTRODUCTION

Endoscopic submucosal dissection (ESD) has revolutionized the treatment of gastrointestinal lesions, allowing for minimally invasive resection of early‐stage tumors. Compared with surgical resection, ESD is less invasive. Compared with piecemeal endoscopic mucosal resection, which can have a local recurrence rate as high as 20%^1^ ESD enables en bloc resection with precise histologic diagnosis (Supporting information).

However, the procedure is technically challenging, and special modifications must be considered in difficult parts of the body as well as with complex lesions. In areas such as the pharynx and the duodenal bulb, complete resection with a standard gastroscope may be challenging due to limited working space.^2^ ESD of esophageal lesions encompassing more than half of the circumference is associated with more difficulty.^3^

Recent case reports have highlighted the potential of a novel slim gastroscope, the EG‐840TP, in overcoming these challenges. This scope has been successfully used for ESD in cases involving severe fibrosis, chemoradiotherapy‐induced strictures, and lesions in anatomically challenging locations such as the pyloric ring.^4^, ^5^, ^6^ The EG‐840TP, with its thinner outer diameter of 7.9 mm and improved down angle capability of 160°, has demonstrated enhanced maneuverability in narrow spaces while maintaining a 3.2 mm accessory channel for standard ESD devices. In this case series, we present our experience with the use of this novel slim gastroscope for ESD in especially challenging lesions, building upon these recent reports and further exploring its potential in complex ESD procedures.

### Patients and methods

We conducted a retrospective analysis of a prospectively maintained de‐identified database of patients who underwent ESD for pharyngeal, esophageal, gastric, duodenal, and rectal lesions at a single tertiary care center between November 2022 and July 2023. A total of 17 patients were included in the study. All procedures were performed using the novel slim gastroscope, EG‐840TP (Fujifilm).

Exclusion criteria included contraindications to general anesthesia, patients under 18 years old, and suspected deep submucosal invasion. Lesions located beyond the reach of this gastroscope, such as those in the proximal colon and duodenum beyond the ampulla, were excluded. All procedures were performed by expert endoscopists with at least 100 ESD procedures.

### Pre‐procedure evaluation

Patients underwent pre‐procedure endoscopy, including white light and image‐enhanced inspection of lesions prior to resection. For ESD in the duodenum, patients received an antispasmodic agent. Colonic ESD procedures were preceded by standard bowel preparation, and patients received an antispasmodic during the procedure.

### Scope design

**FIGURE 1:**
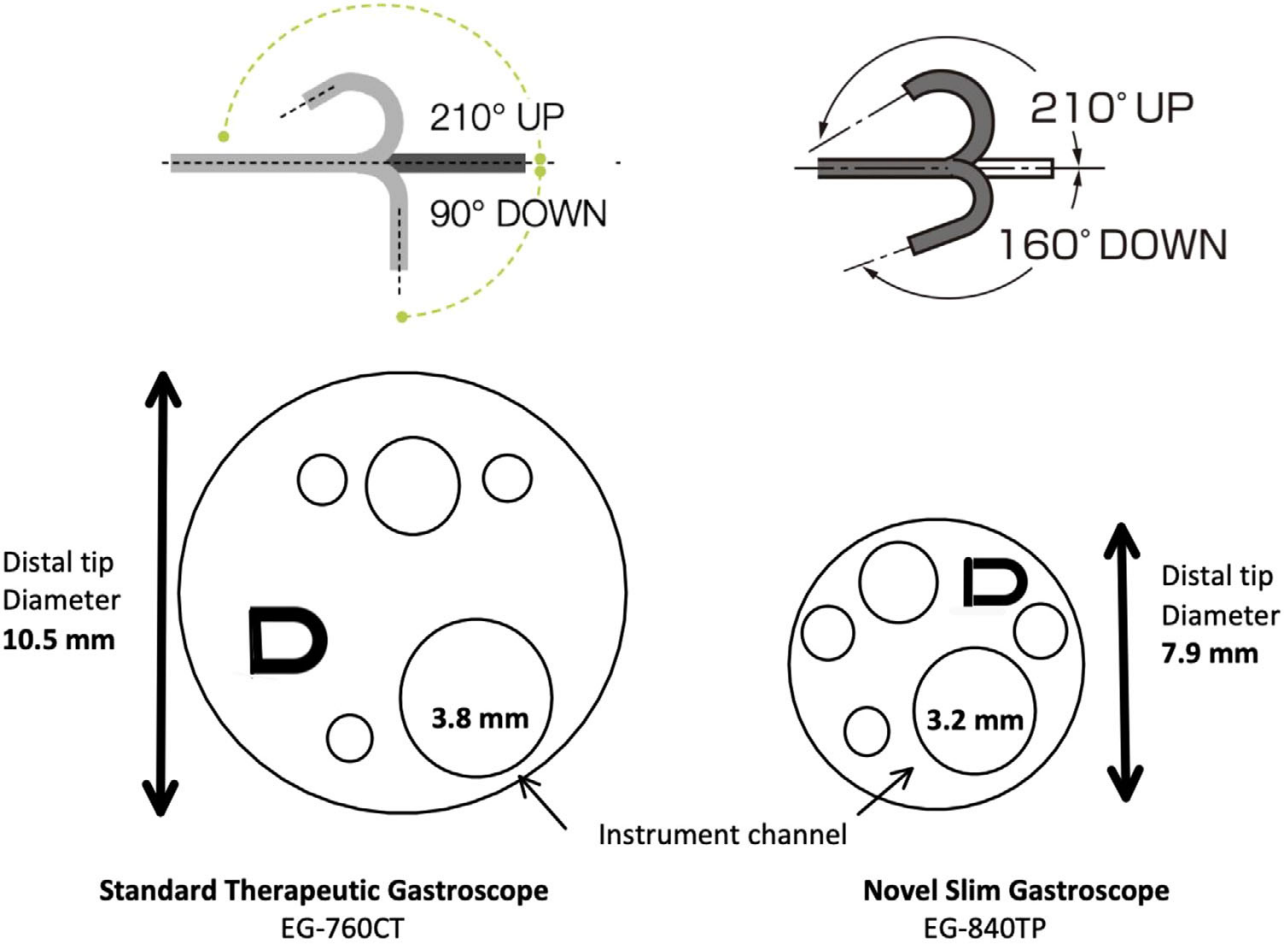
Diagram of the slim scope compared with the standard therapeutic gastroscope.

All procedures utilized the EG‐840TP slim gastroscope (Fujifilm, Tokyo, Japan). This novel device combines a reduced outer diameter of 7.9 mm with a standard 3.2 mm accessory channel. It features a 160‐degree down angle function, enhanced maneuverability, and advanced imaging capabilities including high‐definition white light and narrow‐band imaging. The scope maintains similar ergonomics and length to conventional gastroscopes (Table 1, Figure 1).

**TABLE 1:** Scope specifications.

	Standard therapeutic gastroscope (EG‐760CT)	Novel slim gastroscope (EG‐840TP)
Field of view	140°	140°
Observation range	2–100 mm	2–100 mm
Bending capability	Up 210°/down 90° Right 100°/left 100°	Up 210°/down 160° Right 100°/left 100°
Distal end diameter	10.5 mm	7.9 mm
Flexible portion diameter	10.8 mm	7.9 mm
Working channel diameter	3.8 mm	3.2 mm
Working length	1100 mm	1100 mm
Total length	1400 mm	1400 mm

### Device and accessories

Equipment included a T Tech Knife 1.5 mm (Micro‐Tech), a clear small‐caliber distal transparent cap (Fujifilm), and coagulation forceps (Coagrasper G, FD‐412LR; Olympus). An electrosurgical generator VIO300D (ERBE Elektromedizin Ltd) was used with “EndoCut mode I” (effect 2, duration 2, interval 2) for mucosal incision and “forced coagulation mode” (effect 2, 45 W) for dissection and vessel coagulation. RAICHO2 hemostasis forceps (Kaneka Medics) were employed for hemostasis.

## PROCEDURE

### Pharynx

ESD procedures using the novel slim gastroscope were performed in the operating room under general anesthesia, with patients in the supine position. To optimize working space, the anesthesiologist secured the laryngoscope to the ventral side of the endotracheal tube while viewing the endoscopic image, directly elevating the larynx. Standard ESD technique was employed, with resected lesions retrieved via suction into the distal attachment. In one case, three separate lesions were removed to create a single defect. Post‐procedure, all patients remained intubated for 24 h.

### Esophagus

The patients were placed in the left lateral position. Prior to endoscopy, patients swallowed a defoamer solution consisting of a mixture of pronase, baking soda, dimethicone, and water and gargled with lidocaine. Patients received moderate sedation. The margins of the lesion were confirmed using chromoendoscopy with Lugol's iodine solution and marked for resection. The lesions included in this study had circumferential involvement of the esophageal lumen. Hence, ESD was performed using the multiple tunnel method. Three tunnels were created before performing lateral dissection for a complete connection and en bloc resection was achieved. Triamcinolone was injected into the ulcer bed to prevent stricture formation.

### Stomach

The patients were placed in a left lateral position and sedated with moderate sedation. Pre‐procedure, they swallowed a defoamer solution and gargled it with lidocaine. For some of the gastric cases, standard ESD technique was employed and in others, the pocket creation method was utilized. This involved an initial mucosal incision on the oral side, followed by the creation of a large submucosal pocket. The pocket was progressively opened from the distal end against gravity. Complete submucosal dissection under the lesion enabled en bloc removal.

### Duodenum

The patients received general anesthesia, were intubated, and placed in the left lateral position. Conventional ESD was carried out by making the initial incision in the distal end and then dissecting proximally. Post‐resection, duodenal defects were primarily closed using an over‐the‐scope clip (OVESCO Endoscopy AG) applied with a standard high‐definition endoscope (Olympus GIF‐H290T; Olympus Medical Systems Co. Ltd). The slim scope was then used to place multiple conventional through‐the‐scope clips for further closure. In one case involving the duodenal bulb, a polyglycolic acid sheet was additionally applied to the defect.

### Rectum

The slim scope was used in the rectum to perform endoscopic intermuscular dissection (EID). An initial mucosal incision was made on the distal side and submucosal dissection was performed in a conventional fashion until an area of severe submucosal fibrosis was encountered seen as a distinct narrowing of the submucosal space. The mucosal incision was then created on the proximal side and submucosal dissection was performed around the area of fibrosis. Careful myotomy was then performed in the plane between the longitudinal and circular muscle layers. The lateral sides were cut last and the defect was carefully examined for signs of perforation. Through‐the‐scope clips were placed at the center of the defect, prophylactically, at the site of myotomy.

### Outcomes

Procedural outcomes included technical success, R0 resection rates (negative deep and lateral histologic margins), curative resections (R0 resections with low‐risk tumor characteristics, avoiding further treatment), and histological features (final histology, depth of invasion, grade of differentiation, and presence of lymphovascular invasion).

Resections were defined as en bloc if the lesion was resected in a single piece. The procedure duration (min) was defined as the time between the first endoscope insertion and to final endoscope removal. Specimen size was determined by the largest diameter after stretching onto the cork. Complications were defined as follows: perforation was a complete muscular hole during treatment or clinical evidence of perforation on postoperative computed tomography; bleeding was clinical evidence of post‐ESD bleeding requiring endoscopic treatment.

### Data collection/statistics

Demographic data were collected for each patient, including ASA score, age, sex, and prior attempts at therapy. Quantitative variables are presented as median and range, and qualitative variables by frequencies and percentages. Descriptive statistics were used to analyze the outcomes.

## RESULTS

The cohort comprised 17 patients (four women [24%] and 13 men) who underwent ESD for 20 lesions (Table 2). One patient had three pharyngeal lesions removed as a single defect. Defect sizes ranged from <10 to >40 mm, with the majority (49%) between 20 and 40 mm. En‐bloc and R0 resection were achieved in 100% of cases, with no significant adverse events reported. The median lesion size was 15 mm (range: 2–40 mm), and the median procedure time was 30 min (range: 5–105 min). Various strategies including multiple tunnels, pocket creation, tunnel method, clip and line traction, and EID were employed to achieve en bloc resection.

**TABLE 2:** Patient and lesion characteristics.

Characteristics	
Sex, male, *n*/*N* (%)	13/17 (76%)
Age, median (IQR), years	60 (40‐79)
Lesion size, mean	12 mm (2–40 mm)
Defect size, n (%), mm	
<10 mm	2 (11%)
10–20 mm	3 (16%)
20–30 mm	4 (22%)
30–40 mm	5 (27%)
>40 mm	4 (22%)
Lesion location, *n*/*N* (%)	
Pharynx	6/20 (30%)
Esophagus	5/20 (25%)
Stomach	5/20 (25%)
Duodenum	3/20 (15%)
Rectum	1/20 (5%)
Mean procedure time, min	30 (5–105)
Technical success	17/17 (100%)
R0 resection	17/17 (100%)

## DISCUSSION

**FIGURE 2:**
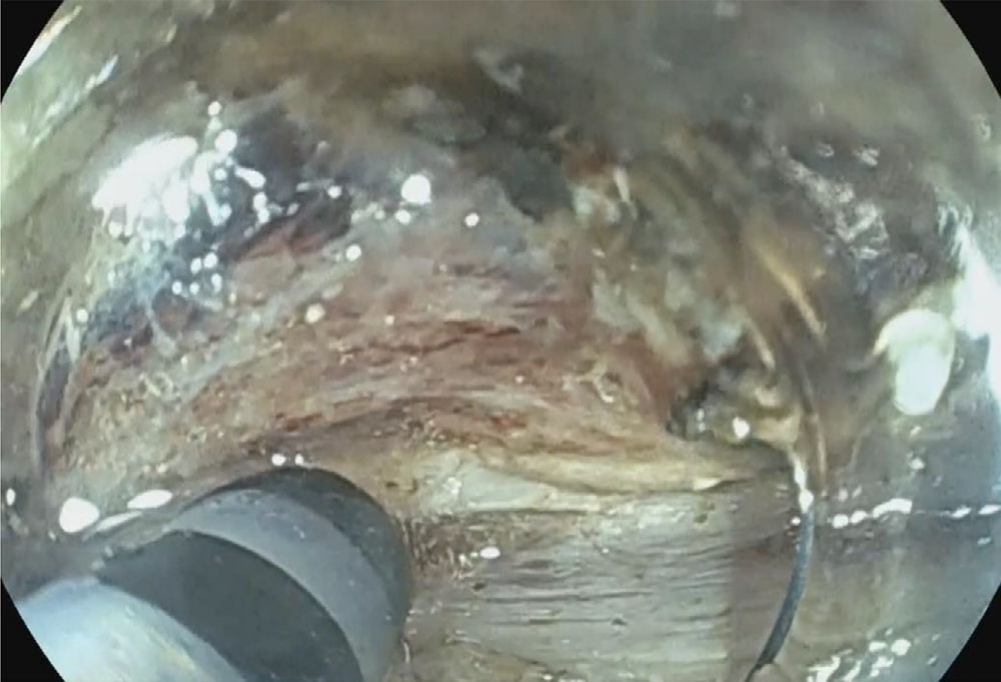
Image of the video.

In this case series, we demonstrated the unique uses of the novel slim gastroscope in various anatomic locations and with specific techniques of ESD (Figure 2). Our experience presents several important findings and raises valuable points for discussion.

Pharynx and duodenum: Pharynx and duodenum present unique challenges for ESD due to limited space and visualization. Our previous pharyngeal ESD study reported a mean procedure time of 37.8 ± 28.2 min, while with the novel slim scope, it averaged 9 min.^1^ Larger studies have shown 59% R0 resection rates in pharyngeal ESD,^2^ compared to our 100% with the slim scope.

Duodenal ESD is challenging due to anatomical factors, including retroperitoneal fixation, distance from the oral cavity, and thin walls prone to perforation. The difficulty in approaching lesions tangentially further complicates the procedure. Proximal duodenal Brunner's glands and fibrosis from biopsies add to the complexity. Kato et al. highlighted the need for advances to standardize duodenal ESD.^4^, ^7^ Recent studies report R0 resection rates of 53%–63%^6^, ^8^ and complication rates of 13% in Western centers and 6.8% in Eastern centers.^6^, ^9^, ^10^ Our study achieved 100% R0 resection without complications, potentially due to the slim scope's improved access and maneuverability.

These results suggest the novel slim scope may enhance outcomes in challenging ESD locations, though larger studies are needed for confirmation.

Benefits in the stomach: This study demonstrates the slim scope's efficacy in resecting large gastric lesions using both pocket creation and conventional ESD techniques. The pocket creation method, introduced by Miura et al. for gastric neoplasm dissection,^11^ is particularly beneficial for large gastric lesions where maintaining tissue tension and adequate submucosal exposure are crucial.

The slim scope's narrow diameter facilitates easier navigation in the stomach, particularly during initial insertion. However, its effectiveness within the pocket itself may not necessarily be superior to conventional scopes. The pocket creation method involves forming a submucosal pocket under the lesion through a small mucosal incision, just large enough to insert the endoscope for submucosal dissection.

While the slim scope shows promise for gastric ESD, further studies are needed to determine the optimal scope for the pocket creation method in the stomach. This research contributes to the ongoing efforts to improve techniques for resecting large gastric lesions, an area that remains technically demanding.

Esophageal ESD: Circumferential ESD for early esophageal cancer is a tedious procedure. ESD using a tunneling technique as well as with a circumferential incision has been analyzed to have a mean procedure time of 118.7 and 102.4 min, respectively using a standard gastroscope.^12^ Using the slim scope, our mean procedure time for ESD of circumferential esophageal lesions was 56 min.

This reduction in procedure time is promising, but factors such as lesion characteristics and operator experience must be considered. Larger studies are needed to confirm these results and assess long‐term outcomes and complication rates.

Rectal lesions: To maximize the chance of achieving complete R0 resection, EID may be necessary for lesions with deep submucosal invasion. EID was first described by Rahni et al. in 2017 for the endoscopic resection of a rectal tumor with severe fibrosis in a patient who refused surgery. Using the double tunneling method, a myotomy was performed through the internal circular muscle layer, creating a dissection plane between the internal circular muscle layer and the external longitudinal muscle layer, and a myectomy was completed. This procedure successfully achieved the en‐bloc resection of a T1b adenocarcinoma with 4350 µm invasion into the submucosa.^13^ This novel, rectal preserving technique evaluated in a prospective study from the Netherlands found that curative resection was achieved in 45% of patients with an overall technical success rate of 96% and R0 resection rate of 81%.^1^ For specific patients with rectal cancers with optical suspicion of deep submucosal invasion but those lacking high‐grade tumor budding, lymphovascular invasion, or poor differentiation, the low rate of lymph node metastasis (1.3%–2.5%) allows for consideration of EID in a rectal wall sparing procedure.^14^, ^15^ EID has an inherent learning curve, and for successful R0 resection, the endoscopist must perform a selective myotomy of the circular muscularis propria, exposing the longitudinal muscle fibers and dissecting within the intermuscular space. The slim scope is particularly advantageous for this procedure as the space for dissection is small and requires precise discrimination of structures. In the study by Moons et al, the mean procedure time was 110 min.^16^ With the slim scope, the EID procedure was completed in 30 min.

Need for further Investigation: This study is the first to assess the feasibility, safety, and technical success of using the novel slim gastroscope, EG‐840TP, for ESD in challenging locations and situations. The EG‐840TP stands out for its large accessory channel, accommodating conventional knives and coagulation forceps. Larger comparative studies are necessary to validate its technical efficacy against other scopes, particularly in difficult areas like the pharynx and duodenum, and in specific scenarios such as circumferential esophageal lesions, large gastric lesions, and rectal lesions with deep submucosal invasion or fibrosis.

### Operator experience and technical considerations

The three operators noted distinct advantages and limitations across different anatomical locations:

In the pharynx, the slim scope's reduced diameter (7.9 vs. 10.5 mm) significantly improved visualization and maneuverability in the confined space, particularly when working with the laryngoscope. The shorter procedure times (average 9 min) compared to historical experience with standard scopes reflect this improved handling.

For circumferential esophageal lesions, operators found the slim scope particularly advantageous during multiple tunnel creation, where its reduced diameter facilitated easier navigation between tunnels. This was reflected in shorter procedure times (average 56 min) for these complex cases.

In duodenal cases, the increased downward bending angle (160° vs. 90°) and enhanced maneuverability helped achieve en bloc resection, though operators noted the scope's decreased stiffness required more careful handling to maintain stable positioning.

For gastric lesions, while the slim scope's reduced diameter aided initial insertion and navigation, operators noted that its increased flexibility sometimes compromised stability during pocket creation, particularly in retroflection. This trade‐off between maneuverability and stability suggests that standard scopes may remain preferable for routine gastric ESD.

In the rectal case involving EID, the slim scope's precise control in confined spaces proved beneficial, though again with the noted limitation of reduced shaft stiffness.

This case series demonstrates the feasibility of using a novel slim gastroscope for ESD in various challenging gastrointestinal locations, including the pharynx and duodenum, as well as for complex lesions such as large gastric lesions, rectal lesions with deep submucosal invasion, and circumferential esophageal lesions. The slim scope's reduced diameter and large working channel appeared to improve access and maneuverability in these difficult areas without compromising tool use.

Our preliminary observations suggest potential benefits in certain scenarios, such as improved visualization in the pharynx and apparently reduced procedure times for circumferential esophageal lesions compared to our historical experience. However, it is crucial to emphasize that our small sample size precludes drawing definitive conclusions about efficacy or safety. These findings should be considered hypothesis‐generating rather than conclusive.

The limitations of this case series include its small sample size, lack of a control group, and potential selection bias. Our comparative observations with historical data are purely descriptive and not based on statistical analysis. Therefore, while our experience suggests potential advantages of the slim scope in specific challenging ESD scenarios, these preliminary findings require validation through larger, well‐designed comparative studies to accurately assess its efficacy, safety, and potential role in clinical practice.

## CONFLICT OF INTEREST STATEMENT

None.

## ETHICS STATEMENT

Approval of the research protocol by an Institutional Reviewer Board. N/A.

## PATIENT CONSENT STATEMENT

N/A.

## CLINICAL TRIAL REGISTRATION

N/A.

## Supporting information




**VIDEO S1** Video including ESD of lesions in the pharynx, stomach, duodenum, and rectum performed with the novel slim gastroscope that highlights the scope's unique features.
