# Supplementary material for: Feasibility of a novel slim gastroscope for endoscopic submucosal dissection: A case series (with video)
Source: DEN Open. 2025 May 6;6(1):e70139. doi: 10.1002/deo2.70139 (PMC12053923; doi:10.1002/deo2.70139)
Supplement: Supplementary file 1 — VIDEO S1 Video including ESD of lesions in the pharynx, stomach, duodenum, and rectum performed with the novel slim gastroscope that highlights the scope's unique features. [file DEO2-6-e70139-s001.docx]

**Video link**

[**https://youtu.be/AmPlj7KBfvE**](https://youtu.be/AmPlj7KBfvE)

**Note:**

1. The video link will be only used for peer review. Therefore, the advertisement which appears at the end of or alongside the video will not be included when the video article is published.

2. Please watch the video in “full screen mode” setting in YouTube. Otherwise, some of the subtitles may not be clearly shown.

**Troubleshooting:**

If you cannot watch the video via the link, please check if you log in to YouTube.

**
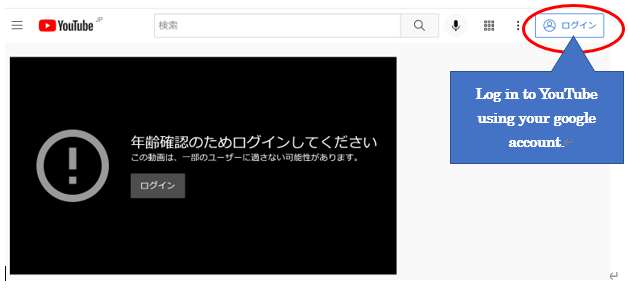
**

In case you cannot watch the video via the link although you logged in to YouTube, please check if your birthday is registered in your google account. For the details, please check the below links.

EN: <https://support.google.com/youtube/answer/3159776?hl=en>

JP: <https://support.google.com/youtube/answer/3159776?hl=ja>
